# Spontaneous resolution of de novo hepatitis B after living donor liver transplantation with hepatitis B core antibody positive graft: a case report

**DOI:** 10.1186/s40792-016-0246-2

**Published:** 2016-10-28

**Authors:** Yasuyuki Hara, Kazuaki Tokodai, Chikashi Nakanishi, Shigehito Miyagi, Naoki Kawagishi

**Affiliations:** The Division of Advanced Surgical Science and Technology, Graduate School of Medicine, Tohoku University, 1-1 Seiryo-machi, Aoba-ku, Sendai, 980-8574 Japan

**Keywords:** De novo hepatitis B, Hepatitis B core antibody positive graft, Living donor liver transplantation, Spontaneous resolution

## Abstract

**Background:**

Hepatitis B core antibody (HBcAb)-positive graft is reported to cause de novo hepatitis B after liver transplantation with a probability of 38–100 % without prophylaxis. Hepatitis B surface antigen loss is reported to be achieved with a probability of only 3–8 % in the patients treated by antiviral agents. We present an extremely rare case of spontaneous resolution of de novo hepatitis B after living donor liver transplantation (LDLT) with HBcAb-positive graft.

**Case presentation:**

An 8-year-old female patient underwent LDLT for end-stage biliary atresia using an HBcAb-positive left lobe graft. After transplantation, she did not receive any prophylactic agents for hepatitis B. Two years after LDLT, she was diagnosed with chronic hepatitis B. Six years after LDLT, liver fibrosis and hepatitis activity were advanced and lamivudine was started. Two years after lamivudine administration, emergence of a lamivudine-resistant YMDD mutant was detected and adefovir dipivoxil was combined with lamivudine. Hepatitis B virus deoxyribonucleic acid (HBV-DNA) became undetectable soon after the addition of adefovir dipivoxil. Twelve years after transplantation, acute rejection occurred and steroid pulse therapy was performed, but hepatitis B did not become severe and HBV-DNA continued to be undetectable. Fifteen years after LDLT, she voluntarily discontinued medication of all drugs, including immunosuppressive agents and antiviral drugs for 1 year because of mental instability. After an interval of 1 year, liver function was normal and her serological HBV status was as follows: HBsAg(−), HBsAb(+), HBeAb(−), HBeAb(+), HBcAb(+) and HBV-DNA(−). From these results, we diagnosed her condition as spontaneous clearance of de novo hepatitis B. The patient is free of antiviral therapies and continues to take a low dose of immunosuppressive drugs and is leading a normal life.

**Conclusions:**

In this case, HBsAg loss is finally achieved but we need to follow carefully for HBV reactivation with the fibrosis of the graft in mind.

## Background

Liver transplantation is the standard treatment for various end-stage liver diseases and acute liver failure. To resolve the organ shortage, hepatitis B core antibody (HBcAb)-positive graft is used in Japan. The HBcAb-positive graft is reported to cause de novo hepatitis B after liver transplantation with a probability of 38–100 % without prophylaxis [1, 2]. The combination of hepatitis B immunoglobulin (HBIG) and nucleoside analogue, such as lamivudine and entecavir, markedly prevents the onset of de novo hepatitis B after liver transplantation using HBcAb-positive grafts [3, 4].

Serum hepatitis B virus deoxyribonucleic acid (HBV-DNA) level is well correlated with the incidence of hepatocellular carcinoma (HCC) [5, 6]. Antiviral therapies, including nucleoside analogue, lead to sustained viral suppression, which leads to the prevention of cirrhosis and HCC [7–9]. Serologic resolution of HBV infection is ideally defined as the loss of hepatitis B surface antigen (HBsAg), seroconversion to hepatitis B surface antibody (HBsAb), and undetectable serum HBV-DNA [10], but HBsAg loss is reported to be achieved with a probability of only 3–8 % in the patients treated by antiviral agents [11, 12]. Current antiviral therapies aim for long-term virological control, and serologic resolution of HBV is rarely achieved.

We report an extremely rare case of spontaneous resolution of de novo HBV after living donor liver transplantation using an HBcAb-positive graft.

## Case presentation

A 91-day-old female patient had undergone Kasai’s operation for biliary atresia (BA). After the operation, liver failure gradually advanced and she was diagnosed with liver cirrhosis at 8 years old. All hepatitis B serological markers before transplantation, including HBV-DNA, HBsAg and HBsAb, were negative (Table 1). The donor was her mother and the donor’s pre-transplant HBV status was as follows: HBsAg(−), HBsAb(+), hepatitis B envelope antigen (HBeAg(−)), hepatitis B envelope antibody (HBeAb(+)) and HBcAb(+).

**Table 1:** Change of hepatitis B serological markers in the clinical course

	Recipient							Donor
Viral marker	Before LDLT	After LDLT	After antiviral therapy	Before anti-rejection therapy	After anti-rejection therapy	Before 1-year cessation	After 1-year cessation	Before donation
HBsAg (IU/ml)	0.29	99.9	>2000.0	>2000.0	146.9	177.6	0.01	0.25
HBsAb (mIU/ml)	0.26	0.0	0.3	0.1	5.0	5.0	19.2	35.46
HBeAg	−	+	+	+	+	+	−	−
HBeAb	−	−	−	−	−	−	−	−
HBcAb	−	+	+	+	+	+	+	+
HBV-DNA (log copies/ml)	<2.6	>7.6	<2.6	<2.6	<2.6	<2.6	<2.1	<2.6

She underwent living donor liver transplantation (LDLT) for end-stage BA using an HBcAb-positive left lobe graft and began an immunosuppression regimen of cyclosporine A (CsA) and steroid. After transplantation, she did not receive HBIG prophylaxis because it was not well known that the HBcAb-positive graft was a risk factor for hepatitis B at that time.

**Fig. 1:**
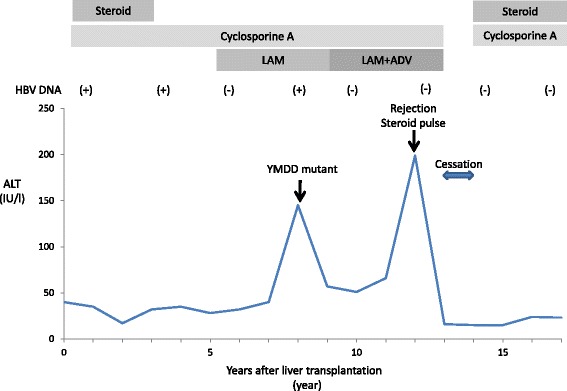
Major events and treatments in the clinical course. The *panel* shows a timeline of the clinical course after liver transplantation. The periods of antiviral agents and immunosuppressive drugs are shown by the *grey bars. LAM* lamivudine, *ADV* adefovir dipivoxil

Two years after LDLT, the hepatitis B markers were changed as follows: HBsAg(+), HBeAg(+), HBeAb(−) and HBV-DNA(+), and she was diagnosed with chronic hepatitis B (Fig. 1). Other liver functions were normal, and she was followed closely without antiviral treatment.

**Fig. 2:**
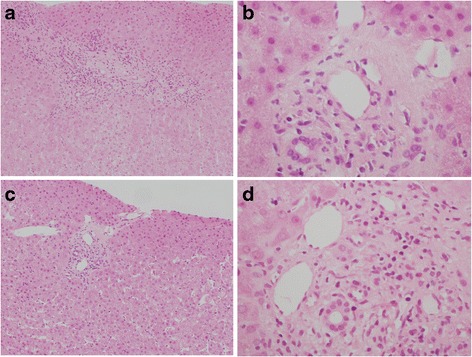
Pathological findings of liver biopsy specimens. **a**, **b** Liver tissue before the treatment of de novo hepatitis B (**a** HE stain ×100, **b** HE stain ×400). Lymphocytic invasion to Glisson’s capsule and piecemeal necrosis were shown. **c**, **d** Liver tissue after lamivudine and adefovir dipivoxil therapy (**c** HE stain ×100, **d** HE stain ×400). Mild invasion of lymphocyte was shown, and liver fibrosis and the activity of hepatitis B were significantly improved

Six years after LDLT, the level of transaminases increased and liver biopsy was performed. From the results of the pathological findings, liver fibrosis and hepatitis activity were advanced (Fig. 2a, b) and lamivudine was started. HBV-DNA became undetectable soon after the start of the antiviral treatment, and the liver function continued to be at a normal level for several years. Two years after lamivudine administration, the serum HBV-DNA level became detectable again and emergence of a lamivudine-resistant YMDD mutant (YIDD) was detected. Adefovir dipivoxil was combined with lamivudine. HBV-DNA became undetectable soon after the addition of adefovir dipivoxil. Liver biopsies, which were performed during the period, showed improvement of the liver fibrosis and hepatitis activity (Fig. 2c, d).

Twelve years after transplantation, acute rejection occurred and steroid pulse therapy (250 mg/day, 3 days) was performed. Hepatitis B did not become severe, and HBV-DNA continued to be undetectable during and after the treatment for acute rejection.

Fifteen years after LDLT, she had obsessive thoughts and suddenly stopped a routine visit and voluntarily discontinued medication of all drugs, including immunosuppressive agents and antiviral drugs for 1 year. During the period, there were no episodes of severe hepatitis and rejection.

She visited our hospital after an interval of 1 year. Mental status was relatively stable and liver function was normal, and her serological HBV status was as follows (Table 1): HBsAg(−), HBsAb(+), HBeAb(−), HBeAb(−), HBcAb(+) and HBV-DNA(−). From these results, we diagnosed her condition as a spontaneous clearance of de novo hepatitis B. The patient is free of antiviral therapies and continues to take a low dose of immunosuppressive drugs (CsA and steroid) and is leading a normal life.

### Discussion

The HBcAb-positive graft is reported to cause de novo hepatitis B after liver transplantation with a probability of 38–100 % [1, 2]. This case received no prophylaxis after transplantation, because it was not well known at the time of liver transplantation that the HBcAb-positive graft was a high risk factor for de novo hepatitis B. Now, in our institution, pre-transplant HBsAg-negative recipients, who received HBcAb-positive grafts, have prophylactic therapies with an injection of high doses of hepatitis B immunoglobulin (HBIG) and administration of nucleoside analogue, and de novo hepatitis B did not occur in two pre-transplant HBsAg-negative recipients using HBcAb-positive grafts who received prophylactic therapies [13].

Viral persistence depends on the balance between viral replication and host immune responses. Immunosuppression regimen of CsA and steroid was continuously given after transplantation, and liver function was normal for 6 years. In this period, viral replication became persistent but active hepatitis might disappear due to host immature immunity and immunosuppressive agents. As the patient grew up, host immunity improved and the immune system might be able to destroy HBV-infected hepatocytes, causing hepatitis. Steroids are a well-known risk factor of HBV reactivation, especially when the dose is equivalent to 20 mg/day [14]. In this case, steroid pulse therapy (250 mg/day, 3 days) was performed for rejection at 18 years after transplantation but HBV reactivation had not occurred, which might be contributed to the inhibition of viral replication by antiviral agents. An acute cellular rejection was reported to have possibilities to evoke antiviral response in cases of hepatitis C virus-positive recipients and might be involved in the spontaneous resolution of HBV [15].

Most initial HBV infections in adulthood result in complete recovery with HBsAg loss and the production of HBsAb. In this case, hepatitis markers had finally changed as follows: HBsAg loss, seroconversion to anti-HBs antibody and sustained suppression of HBV-DNA. Voluntary cessation of immunosuppressive agents resulted in the normalization of host immunity, and the infectious status of HBV was thought to change into the so-called “initial infection in adulthood”. Spontaneous clearance of HBV had happened fortunately without becoming fulminant hepatitis. The administration of antiviral agents until the cessation of drugs suppressed viral replication, which might contribute to the prevention of fulminant hepatitis.

HBsAg loss is rare with a probability of only 3–8 % in the patients treated by antiviral agents [11, 12], and HBsAg levels are reported to correlate well with the intrahepatic covalently closed circular DNA (cccDNA) level [16, 17]. We think that the definition of the spontaneous resolution of de novo hepatitis B is the serological HBV status: HBsAg(−) and HBV-DNA(−). Despite serological resolution, HBV cccDNA remains in hepatocytes and circulating peripheral mononuclear cells [18, 19]. The necessary conditions of the cessation of antiviral therapy were reported [20, 21], and we think that the cessation of antiviral therapy could be performed safely if the serological HBV status is HBV-DNA(−), HBeAg(−) and/or HBsAg(−). This case fulfilled the necessary conditions and stopped antiviral therapy, but a low dose of immunosuppressive drugs was administered in order to prevent rejection and the fibrosis of the graft.

## Conclusions

We present an extremely rare case of the spontaneous resolution of de novo hepatitis B after LDLT with an HBcAb-positive graft. HBsAg loss is finally achieved, but we need to follow the patient carefully for HBV reactivation with the fibrosis of the graft in mind.

## Abbreviations

HBcAb: Hepatitis B core antibody
HBeAg/Ab: Hepatitis B envelope antigen/antibody
HBsAg/Ab: Hepatitis B surface antigen/antibody
HBV-DNA: Hepatitis B virus deoxyribonucleic acid
LDLT: Living donor liver transplantation
